# The association between reaction time variability and social problems in children with ADHD: support for the role of attentional fluctuations in social interactions

**DOI:** 10.1007/s00787-025-02787-6

**Published:** 2025-06-16

**Authors:** Marie Munch, Berge Osnes, Virginia Peisch, Heike Eichele, Elisabet Kvadsheim, Daniel Wollschlaeger, Kerstin J. Plessen, Lin Sørensen, Anne Arnett

**Affiliations:** 1 University of Bergen, Bergen, Norway; 2 Boston Children’s Hospital, Boston, United States; 3 Haukeland University Hospital, Bergen, Norway; 4 University Medical Center of the Johannes Gutenberg University Mainz, Mainz, Germany; 5 University Hospital of Lausanne, Lausanne, Switzerland; 6 Harvard University, Cambridge, United States

**Keywords:** ADHD, Social problems, Reaction time variability, Neurocognition

## Abstract

Children with attention-deficit/hyperactivity disorder (ADHD) typically experience social interaction problems. Neurocognitive functions, such as working memory and inhibitory control have been included in several studies of social problems in ADHD, with inconsistent findings emerging. Intraindividual reaction time variability (RTV) has been understudied as a factor explaining social problems in children with ADHD, despite being one of the most consistent cognitive impairments in the disorder. In the current study, we hypothesized that greater RTV would relate to greater parent-reported social problems and may reflect the negative impact of attentional fluctuations on social interactions in ADHD. We tested the association between RTV and social problems while accounting for the effects of working memory and inhibitory control in two independent samples of children with ADHD and typically developing (TD) peers. The neurocognitive functions were assessed through performance-based laboratory measures. Sample one, derived in Norway, included 41 children with ADHD and 35 TD children. Sample two, derived in the United States, included 73 children with ADHD and 26 TD children. Linear regression analyses, controlling for other relevant variables, indicated that greater RTV was associated with greater social problems across both samples. Our results support the view that attentional variability is linked to social problems in children with ADHD.

**Supplementary Information:**

The online version contains supplementary material available at 10.1007/s00787-025-02787-6.

## Introduction

Children with ADHD typically have problems with social interactions [1] that persist into adolescence [2] and adulthood [3]. Social problems in peer interactions among children with ADHD include fewer reciprocated friendships and more experiences of peer rejection [4]. These challenges are associated with adverse long-term mental health outcomes, including depression and anxiety symptoms [5, 6]. Social problems in ADHD do not seem to be caused by a lack of knowledge or skills; rather, children with ADHD appear to struggle with applying these skills in real-life social interactions [7]. Studies of social problems in ADHD have typically postulated that poorer abilities to monitor and modulate incoming information (i.e., working memory) and suppress a dominant, prepotent response, allowing for controlling attention, thoughts, and behaviors (i.e., inhibitory control), explain social problems. However, few studies have investigated how fluctuating attention may further contribute to social problems. Attention fluctuation can be measured via intraindividual reaction time variability (RTV), with increased RTV being one of the most consistent neurocognitive dysfunctions in ADHD [8–11]. We hypothesized that in social situations, fluctuating attention may lead to missed social information, thereby contributing to increased functional problems. In the current study we test whether RTV explains variance in social problems among children with ADHD in two independent samples.

Inhibitory control and working memory are two neurocognitive functions often studied in relation to social problems [see e.g., 12]. Both play an important role in complex, goal-directed behaviors [13], and are thus good candidates for understanding social interaction problems. Several studies have found that social problems are associated with poorer working memory [12, 14–19]. Poorer inhibitory control, on the other hand, has not been as consistently associated with social problems [see 12, 14, 16, 18, 20, 21].

One aspect of attention that is not specifically measured with inhibitory control or working memory is sustained attention, which is essential for the overview and meta-awareness of relevant social information. RTV during speeded response tasks is suggested to measure moment-to-moment fluctuations in attention [8, 10, 22]. To our knowledge, only two studies have investigated the association between social problems and RTV in children with ADHD [21, 23]. One of these found that lower child-reported social functioning and higher proactive/reactive aggression was associated with greater RTV [23]. The other study did not find that social problems were associated with RTV, inhibitory control, or working memory [21].

## The current study

The current study investigates the neurocognitive correlates of social problems among 8–12-year-old children with ADHD and typically developing (TD) children across two independent samples—one from Norway and one from the USA. Our primary aim is to examine whether reaction time variability (RTV) predicts social problems, a relationship that has received limited attention in previous research and has produced conflicting findings. Specifically, we hypothesize that higher RTV will independently predict more parent-reported social problems. Additionally, we include the more extensively studied neurocognitive functions of working memory and inhibitory control. Based on prior findings, we expect that working memory, but not inhibitory control, will also predict social problems. It is possible that RTV affects another aspect of processing of social information than working memory. For instance, RTV can tap into the attention focus in social situations and working memory the ability to cognitively process and modulate social information in accordance with the specific social situation and its context.

## Sample one methods

### Participants

Sample one participants came from a larger study (*N* = 102) investigating cognitive and emotional development in medication-naïve children aged 8–12, with ADHD and/or Tourette syndrome recruited from psychiatric outpatient clinics in Bergen, Norway, and TD children recruited from local schools in the same municipality [24]. Exclusion criteria for the larger study were full scale IQ (FSIQ) < 75, use of psychostimulants, autism spectrum disorders, prior head injury or epileptic seizures, or gestational age < 36 weeks. All children were evaluated by a psychologist and a psychiatrist. Diagnostic assessments, including assessment of exclusion, were based on available clinical information, the semi-structured interview Kiddie Schedule for Affective Disorders and Schizophrenia–Present and Lifetime Version [K-SADS-PL; 25,26], WISC-IV [27], The Children Global Assessment scale [28], and a DSM-IV ADHD rating scale [29]. Twenty-six children with a diagnosis of Tourette syndrome were excluded from these analyses. A such, the final sample for the current study included 41 children with ADHD and 35 TD children. The ADHD sample was 29% female, and the TD sample 42% female (χ^2^ [1] = 0.986, *p* =.320). The groups did not differ in age (ADHD *M* = = 10.18, *SD* = 1.29 years; TD *M* = = 10.04, *SD* = 1.08; *t*[73] = 0.53, *p* =.593). No data regarding ethnicity or race was collected because such data collection would have violated Norwegian privacy and data protection laws.

### Procedures

All study procedures were approved by the Regional committee for medical research ethics of western Norway (study number: 2014/1304) and research was conducted according to relevant regulations and guidelines. Children were accompanied by their caregivers to the research visit, which included psychosocial and diagnostic assessment and neurocognitive testing over two consecutive days. Informed written consent and assent were obtained from participants and their proxies at the beginning of the visit. The duration of the neurocognitive testing was approximately three hours.

### Measures

#### Cognitive ability

The Wechsler Intelligence Scale for Children, Fourth Edition [27] was administered to all children to derive FSIQ.

#### ADHD symptom severity

A primary caregiver completed the DSM-IV ADHD rating scale [29], which measures frequency of the 18 ADHD symptoms of inattention and hyperactivity/impulsivity in children. Sample items include “Has difficulty sustaining attention in tasks or play activity” (inattentive domain) and “Has difficulty awaiting turn” (hyperactive/impulsive domain). All items were rated on a four-point Likert scale, with higher scores indicating more ADHD symptoms. For each participant, a total ADHD symptom severity score was computed by summing the ratings across all 18 items. The scale has high cross-cultural validity and reliability [30,31

### Inhibitory control

The third condition of the Delis-Kaplan Executive Functioning Scales Color-Word Interference Test [D-KEFS CW3; 32] was used to measure inhibitory control, as it captures cognitive inhibition and interference control relevant for navigating complex social interactions. The child is instructed to name the ink-colors of a set of printed color words, as quickly as possible. The ink colors are incongruent with the words (e.g., the word ‘blue’ is printed in green, and the correct response is “green”), thus requiring the child to override the prepotent response to read the word. The current study used the CW3 total error score, as it is a more sensitive measure of inhibitory control than the CW3 response time score in ADHD samples [33].

#### Working memory

The Letter Number Sequencing task (LNS), from the WISC-IV [27] was used to assess working memory. In this task, the child hears a series of letters and numbers (with increasing length) and is asked to repeat the sequence back to the examiner in a particular order. The LNS is a common and established measure of verbal working memory capacity, and the clinical measure most highly associated with laboratory assessments of working memory [34,35]. To align with Sample two, we also included the Digit Span Backward task (DSB) from WISC-IV [27]. In this task the instruction is to repeat a series of numbers, presented verbally, in the reverse order. LNS and DSB scores were analyzed separately.

#### Intraindividual reaction time variability (RTV)

RTV and mean reaction time (RT) were derived from the Stop Signal Test (SST), a computer-based task from the Cambridge Neurocognitive Test Automated Battery (www.cambridgecognition.com/cantab). The SST consisted of 320 test trials, where 25% of the trials were stop trials and 75% were go trials (see [24]). The coefficient of intraindividual reaction time variability RTcv, was used as a measure of RTV, calculated as the standard deviation of reaction time (SDRT) divided by the mean reaction time (MRT) [8, 10]. To account for potential right skewness of the reaction time distributions, an ex-gaussian RT calculation of *tau* (τ), was derived and included in a follow up analysis [e.g. see 8]. *Tau* is a measure of the exponential component of the RT distribution, which can also be explained as the mean of longer reaction times [36].

#### Social problems

A primary caregiver completed the Child Behavior Checklist 6–18 [CBCL; 37]. The age- and sex-standardized *T*-score (*M* = 50, *SD* = 10) on the CBCL Social Problems subscale was used to measure social problems, with higher scores reflecting more social problems. The Social Problems subscale includes 11 items (e.g., “Gets teased a lot” or “Not liked by other children”) rated on a three-point Likert scale. The subscale has high test-retest reliability (0.90) and a Cronbach’s alpha of 0.82 [37].

## Sample two methods

### Participants

Sample two recruitment and procedures have been described previously [38]. One-hundred forty one 7–11-year-old children with and without ADHD were recruited from clinics and community settings in the greater Seattle, WA area USA. ADHD diagnoses were confirmed by a licensed clinical psychologist using a combination of clinical interview with a primary caregiver, review of caregiver ratings on an ADHD rating scale and on the CBCL, review of medical records when available, and direct observation of the child during the research visit. Exclusion criteria were assessed through parent-report, with IQ confirmed by testing during the research visit, and concerns for autism spectrum disorder ruled out by a licensed child psychologist through observation. Following enrollment, 120 children met inclusion criteria, including confirmed ADHD diagnosis or TD status, FSIQ ≥ 80, lack of perinatal trauma, and absence of autism spectrum disorder, intellectual disability, or seizures. To align with Sample one, children younger than 8 years old were excluded from the current analyses. The final sample for the current analyses therefore included 73 children with confirmed ADHD and 26 TDs. The male to female ratio of enrolled participants was designed to approximate epidemiological rates for ADHD [39]; in the end, 30% of the ADHD sample and 35% of the TD sample were female (χ [1] = 0.03, *p* =.860). The groups did not differ in age (ADHD *M* = 10.1, *SD* = 1.10 years; TD *M* = 9.66, *SD* = 1.10 years; *t*[97] = 1.76, *p* =.082). 11% of the sample identified as Hispanic/Latino. 72% of the sample identified as European/White; 20% as multiracial; 3% as African American/Black; 3% as Asian/Pacific Islander; 1% as Native American/Indian; and 1% declined to answer.

### Procedures

Participants visited the laboratory with a caregiver for a three-hour visit that included neurocognitive testing. Parents consented to their child’s participation and children assented, consistent with the institutional review board (IRB) approved procedures of the governing institutional review board (IRB) at the University of Washington. Children who were prescribed medications for ADHD (64%) abstained from taking stimulants for at least 48 h prior to the research visit, or non-stimulants for a washout duration determined by the prescribing provider. When applicable, caregivers were instructed to rate their child’s un-medicated behavioral and psychiatric symptoms.

### Measures

#### Cognitive ability

Cognitive ability (i.e., FSIQ) was measured using the 2-subtest version (i.e., Vocabulary and Matrix Reasoning) of the Wechsler Abbreviated Scale of Intelligence, Second Edition [WASI-II; 40].

#### ADHD symptom severity

ADHD symptoms were assessed using the Strengths and Weaknesses of ADHD Symptoms and Normal Behavior Scale [SWAN; 41]. Caregivers were asked to compare their child’s behavior to that of other same-aged children on a 7-point Likert scale. Specifically, caregivers were asked to evaluate their child’s behavior by providing ratings ranging from *Far Below* to *Far Above* other children of the same age. Mean severity scores were derived for inattention (items 1–9) and hyperactivity (items 10–18) such that higher scores reflected greater impairment.

#### Inhibitory control

Inhibitory control was measured as described in Sample One.

#### Working memory

Working memory was assessed using the digit span backward subtest from the WISC-V [42]. Please see Sample One Measures for further description.

#### Intraindividual reaction time variability

RT and RTV were derived from an adapted stop signal task described previously [43]. Briefly, this task included 64 trials of a visual forced-choice response task, wherein children were instructed to press one key in response to presentation of an “X” and another key in response to presentation of an “O.” The stimulus duration was 500 ms, with a random interstimulus interval ranging from 1030 to 1050 ms. Next, an additional 64 trials of the task were completed, this time with an auditory “stop” signal (1000 Hz tone with a duration of 250 ms) randomly presented prior to 25% the visual stimuli. Children were instructed to withhold their response when they heard the sound. The timing of the stop signal was adjusted for each trial according to the child’s performance on the previous stop trial. For the current study, only trials without a stop signal and trials with response times > 100 ms were included. Mean RT and RTV (standard deviation of RT) were computed and standardized within each task (forced choice versus stop-signal), then averaged across tasks for each individual.

#### Social problems

Primary caregivers reported on the Social Problem Scale from the Child Behavior Checklist [37], consistent with Sample One.

### Statistical analyses for sample one and sample two

All statistical analyses were conducted with R [41]*tau* was included in separate regression analyses. Furthermore, we also run supplementary analyses replacing our measure of inhibitory control (D-KEFS CW3) with SSRT, a measure of the behavioral aspects of inhibition. To correct for multiple testing, we applied a Bonferroni adjustment to our two main regression models (RTV and tau), setting the significance threshold at α = 0.05/2 = 0.025.

## Results

### Descriptive statistics

All variables showed normal distributions with skew <|2.50| and kurtosis < 5.0. Across samples, children with ADHD had more parent-reported social problems, increased RTV, reduced working memory scores and reduced IQ scores compared to TDs. In sample one only, children with ADHD displayed reduced inhibitory control compared to TDs (Table 1).

**Table 1:** Descriptive statistics for main study variables by diagnostic group

Sample One			
Variable	ADHD (*n* = 41)	TD (*n* = 35)	*p*
Social Problems	60.58 (8.56)	50.94 (1.92)	**< 0.001**
RTV	0.39 (0.11)	-0.42 (0.58)	**< 0.001**
Tau	173.09 (83.42)	122.47 (43.97)	**0.002**
RT	0.21 (1.)	-0.23 (0.86)	0.062
Inhibitory Control (CW3 error)	7.32 (3.42)	9.54 (3.39)	**0.006**
Inhibitory Control (SSRT)	0.18 (1.17)	-0.19 (0.74)	0.110
Working memory (LNS)	6.78 (2.54)	9.57 (3.17)	**< 0.001**
Working memory (DSB)	8.51 (1.93)	9.57 (2.94)	**0.027**
FSIQ (WISC-IV)	91.46 (7.51)	105.83 (10.92)	**< 0.001**
Age	10.18 (1.29)	10.04 (1.03)	0.594
Sample Two			
Variable	ADHD (*n* = 73)	TD (*n* = 26)	*p*
Social Problems	61.85 (8.68)	52.42 (3.44)	**< 0.001**
RTV	0.15 (0.87)	-0.42 (0.70)	**0.002**
Tau	128.60 (45.49)	105.71 (36.97)	**0.021**
RT	-0.16 (0.88)	-0.08 (0.72)	0.674
Inhibitory Control (CW3 error)	5.59 (4.14)	3.96 (3.38)	0.057
Inhibitory Control (SSRT)	0.30 (0.15)	0.30 (0.22)	0.940
Working Memory (DSB)	9.32 (2.85)	10.81 (2.28)	**0.011**
FSIQ (WASI-II)	109.40 (11.24)	116.5 (10.16)	**0.005**
Age	10.10 (1.10)	9.66 (1.10)	0.087

Note. TD = Typically developing; RTV = Reaction time variability (z); RT = Mean reaction time (z); Tau = RT distribution tail; CW3 = D-KEFS Color-Word Interference Test, Condition 3; SSRT = Stop signal reaction time; DSB = Digit Span Backward; LNS = Letter Number Sequencing; FSIQ = Full Scale IQ; WISC-IV = Wechsler Intelligence Scale for Children–IV; WASI-II = Wechsler Abbreviated Scale of Intelligence–II

Social problems were significantly correlated with symptoms of hyperactivity/impulsivity and inattention in both samples. RTV correlated significantly with inattention and hyperactivity/impulsivity in both samples. Older age was associated with increased social problems and decreased RTV in sample one only, and with decreased RT in both samples (Table 2).

**Table 2:** Bivariate correlations between main study variables

Sample One									
Variable	1	2	3	4	5	6	7	8	9
1. Social Problems									
2. RTV	0.22								
3. Tau	0.13	0.80***							
4. RT	− 0.01	0.53***	0.85***						
5. Inhibitory Control	0.07	0.27*	0.28*	0.26*					
6. WM (DSB)	− 0.17	− 0.12	− 0.17	− 0.09	− 0.34**				
7. WM (LNS)	− 0.41***	− 0.20	− 0.28*	− 0.21	− 0.32**	0.48***			
8. Hyp/Imp	0.65***	0.44***	0.37**	0.25*	0.32**	− 0.26*	− 0.37**		
9. Inattention	0.68***	0.50***	0.42***	0.29*	0.28*	− 0.22	− 0.42***	0.86***	
10. Age	0.23*	− 0.32**	− 0.36**	− 0.38***	− 0.11	− 0.09	− 0.04	0.07	0.03
Sample Two									
Variable	1	2	3	4	5	6	7	8	9
1. Social Problems									
2. RTV	0.25*								
3. Tau	0.22*	0.57***							
4. RT	-0.06	-0.29	-0.02						
5. Inhibitory Control	-0.05	0.07	-0.03	0.12					
6. WM (DSB)	-0.05	-0.22	0.04	0.04	-0.15				
7. Hyp/Imp	0.45***	0.36**	0.27	0.04	0.16	-0.27			
8. Inattention	0.47***	0.33*	0.30	-0.10	0.14	-0.31	0.75***		
9. Age	-0.05	0.04	-0.01	-0.34	-0.27	-0.11	0.08	0.21	

Note. RT = Reaction time; RTV = Reaction time variability; DSB = Digit Span Backward; LNS = Letter Number Sequencing; Hyp/Imp = Hyperactivity/Impulsivity. Values are Pearson correlations. ***p <.001, **p <.01, *p <.05

### Linear regressions

The regression analysis for sample one, including RTV, RT, inhibition, working memory (LNS), and age, was significant: *F*(5,66) = 5.01, *p* <.001, adjusted *R*^2^ = 0.22. Higher levels of social problems were associated with greater RTV and lower working memory performance. Older age was also associated with more social problems (Table 3). When DSB was applied as the working memory measure instead of LNS, only RTV and age was associated with social problems (See Online Resource 1).

The regression analysis for sample two with the predictors mean RT, RTV, working memory, inhibitory control, and age was not statistically significant: *F*(5,82) = 1.65, *p* =.157, adjusted R2 = 0.04. However, greater RTV was significantly associated with social problems over and above the other independent variables (Table 3). When SSRT was applied as the measure of inhibition, i.e., behavioral inhibition, the regressions yielded similar results as with D-KEFS CW3 (See Online Resource 2).

The models using the ex-Gaussian measure of tau, as opposed to RTV, yielded similar results. In Sample One, the model was significant, *F*(5, 66) = 4.53, *p* <.001, adjusted R² = 0.20. However, in Sample Two, the model was not significant, *F*(5, 75) = 1.40, *p* =.233, adjusted R² = 0.03. Notably, tau, like RTV, was associated with social problems in both samples.

**Table 3:** Regression table for samples one & two with social problems as the dependent variable

	Sample One					Sample Two				
**Models with RTV** Variable	Raw B	SE	Std B	*t*	*p*	Raw B	SE	Std B	*t*	*p*
RTV	2.48	0.99	18.91	2.52	0.014	2.56	1.15	0.25	2.23	0.029
RT	-1.21	1.00	-2.04	-1.21	0.229	-0.56	1.23	-0.05	-0.45	0.653
Inhibitory Control	-0.13	0.19	-0.31	-0.66	0.512	-0.16	0.24	-0.07	-0.66	0.508
WM (LNS)	-0.93	0.27	-6.00	-3.42	0.001					
WM (DSB)						-0.31	0.37	-0.09	-0.84	0.403
Age	1.54	0.74	11.90	2.08	0.042	-0.61	0.93	-0.08	-0.66	0.510
**Models with Tau**										
Tau	0.05	0.02	0.36	2.11	0.039	0.06	0.05	0.23	2.05	0.044
RT	-2.85	1.58	-4.81	-1.81	0.076	-1.34	1.26	-0.13	-1.06	0.291
Inhibitory Control	-0.08	0.20	-0.19	-0.41	0.684	-0.09	0.26	-0.04	-0.34	0.736
WM (LNS)	-0.89	0.28	-5.69	-3.17	0.002					
WM (DSB)						-0.51	0.39	-0.15	-1.33	0.187
Age	1.42	0.74	0.15	1.90	0.062	-1.69	0.98	-0.09	-0.70	0.484

Note. RTV = Reaction time variability; RT = Reaction time; Tau = Ex-Gaussian RT parameter; WM = Working memory; LNS = Letter Number Sequencing (Sample 1); DSB = Digit Span Backward (Sample 2). Raw B = unstandardized beta; Std B = standardized beta

## Discussion

In the current study, we investigated whether RTV, after adjusting for the independent effects of working memory and inhibitory control, was associated with parent-reported social problems in two samples of children with and without a diagnosis of ADHD. Based on the prior literature, we hypothesized that greater RTV would be associated with greater parent-reported social problems and may reflect the negative impact of attentional fluctuations on social interactions in ADHD. Children with ADHD often have problems with social interactions [1], and the exact reasons for such vulnerabilities are currently not well understood.

Consistent with prior research, we found that children with ADHD had greater parent reported social problems, lower working memory, and greater RTV compared to TDs in both samples. Additionally, inattention and hyperactivity/impulsivity correlated significantly with RTV in both samples. In the regression analyses, we found that greater RTV was associated with social problems after accounting for age, inhibitory control, and working memory in both samples, consistent with our hypothesis. However, the association between reduced working memory for verbal information (when prompts were alphanumeric) and social problems was only found in sample one.

Our results align with a previous study that found a positive association between RTV and children’s self-reported social functioning [23], also when using the ex-gaussian measure of *tau* (i.e., the mean of longer reaction times). RTV, calculated by standard deviation, and *tau* typically correlate highly with one another [45], and increased *tau* in ADHD has been suggested to strengthen the interpretation of RTV as a measure of attentional fluctuations [10]. Greater RTV has, as such, been suggested to reflect attentional lapses that may interfere with processing of social information, such as encoding and interpreting social cues [46]. However, Tamm et al. [23] found no relation between RTV and caregiver ratings of social problems, likely due to methodological differences, including their exclusive use of an ADHD-only sample, which limits variability in social functioning. Additionally, they reported mild ADHD symptoms that did not associate with RTV. Similarly, Sjöwall and Thorell [21] also found no association between RTV and social problems, but their study used a different analytical approach to investigate how ADHD diagnosis predicts social issues and included mediating factors. These differences complicate direct comparisons across studies. A limitation of the current study is that our sample size did not allow for separate group analyses to explore mediation effects within the ADHD and TD groups. Future research should aim for larger samples to better understand how RTV relates to social problems among children with ADHD.

We expected that greater RTV would complement poorer working memory in explaining more parent-reported social problems. This expectation was only confirmed in sample one, which included a measure of alphanumeric verbal working memory (LNS). This finding is consistent with prior studies showing a negative association between poorer working memory on the LNS and greater social problems in children with ADHD [e.g., see 12]. Impaired working memory may affect a child’s ability to keep social goals and social information in mind, while simultaneously planning and executing social responses [17]. Reduced working memory capacity may be more impairing in situations with high working memory demands, such as complex social interactions [47], also specifically in ADHD [see 34]. Similarly, of relevance for social problems in ADHD [see 49], poorer scores on the LNS were found to specifically relate to greater parent-reports of emotional lability in children with ADHD in a prior study [50]. Thus, the finding that RTV and LNS performance each explained variance in social outcomes in Sample one may reflect independent, additive effects of attentional fluctuations or lapses and verbal communication processing problems on social outcomes. Future research is needed to further elucidate the interplay between RTV, working memory, and social problems, as understanding these relationships will enhance our insight into the underlying mechanisms affecting social interactions in children with ADHD.

Inhibition was not correlated with social problems in either sample, consistent with several previous studies [e.g., 18]. Interestingly, a recent study found that while performing a risky decision making task, delay aversion (preference for immediate actions/rewards irrespective of consequences), and not lower inhibitory control scores (shorter decision making response times), predicted longitudinal social problems in ADHD [2]. It remains for future studies to test if delay aversion outside a risky decision-making context would explain variance in social problems beyond attentional fluctuations and working memory in ADHD. Notably, performance on measures of working memory also relies on inhibitory control [51,52] and performance on these tests was correlated in sample one. Thus, it is possible that the link between lower working memory and greater social problems to some degree also reflects impaired inhibitory control.

The current study comprised two independent, samples of children with ADHD. In both samples greater RTV associated with more social problems. There were, however, some differences in the study design in addition to the already-mentioned differences in working memory tasks. The children in the Norwegian sample were medication naïve, whereas some of the children in sample two were being prescribed central nervous system (CNS) medications. Importantly, children in the US sample abstained from taking their ADHD medications for at least 48 h prior to completing research tasks. Despite this difference in medication status, similar results emerged in relation to RTV. Prior research suggests that the positive effects of CNS medication on different cognitive control functions cease when off medication [53], which would be consistent with our results. Similarly, CNS medications may have limited effects on improving social functioning in children with ADHD [54–56], which aligns with the results of the current study in that the children with ADHD had substantially more social problems than the TD peers in both samples.

Further, there were no difference in age and gender distribution between the ADHD and typically controls. In accordance with typical findings in ADHD [11], the children with ADHD had significantly lower general abilities as measured with FSIQ than their typically developing peers. Additionally, the USA sample had a higher average FSIQ compared to the Norway sample, which may have been due to differences in the standardized tests used to measure FSIQ. We opted not to include FSIQ in our regressions because previous research has established that FSIQ and working memory are highly correlated [57, 58].

In sum, the current study supports the hypothesis that attentional fluctuations and verbal working memory are related to social problems among children with ADHD. We propose that these neurocognitive problems may contribute to the social performance difficulties in pediatric ADHD [7], though longitudinal studies are necessary to determine causality. An implication of this finding is that children with ADHD may benefit more from ‘in vivo’ social support and scaffolding, accounting for neurocognitive difficulties, rather than more traditional social skills training [59]. Structuring social settings in a manner that makes them less cognitively overwhelming for the child and provide hands-on social prompting can facilitate interactions and the child’s access to acquired social skills [60].

## Electronic supplementary material

Below is the link to the electronic supplementary material.

Supplementary Material 1

Supplementary Material 2

## Data Availability

Due to reasons of sensitivity/privacy policy, the data from the current study are not openly available. Access to the data can be provided by the corresponding author upon reasonable request.
