# Supplementary material for: The association between reaction time variability and social problems in children with ADHD: support for the role of attentional fluctuations in social interactions
Source: Eur Child Adolesc Psychiatry. 2025 Jun 16;34(12):3843–52. doi: 10.1007/s00787-025-02787-6 (PMC12743078; doi:10.1007/s00787-025-02787-6)
Supplement: Supplementary file 1 — Supplementary Material 1 [file 787_2025_2787_MOESM1_ESM.pdf]

Online Resource 1

To align with Sample 2, we conducted a follow-up regression for Sample 1, replacing the working memory task Letter Number Sequencing (LNS) with Digit Span Backward (DSB). The model including RT, RTV, inhibition, working memory (DSB), and age was significant: F(5,66)=2.37, p=.048, adjusted R^2=0.07. When DSB was applied as the working memory measure instead of LNS, only RTV and age were associated with social problems. See Table S1 for individual predictors.

Table S1 *Regression Table for Sample 1 with Social Problems as the Dependent Variable, including DSB as a working memory measure*

Variable	Raw B	SE	Std B	***t***	*p*
RTV	2.7	1.07	20.54	2.53	.014
RT	-0.8	1.07	-1.36	-0.75	.455
Inhibitory Control	0.01	0.21	0.04	0.06	.954
WM (DSB)	-0.25	0.37	-1.62	-0.68	.498
Age	1.81	0.8	13.94	2.24	.028

Note. RTV = Reaction time variability; RT = Reaction time; WM = Working memory; DSB = Digit Span Backward. Raw B = unstandardized beta; Std B = standardized beta.
