# Supplementary material for: The association between reaction time variability and social problems in children with ADHD: support for the role of attentional fluctuations in social interactions
Source: Eur Child Adolesc Psychiatry. 2025 Jun 16;34(12):3843–52. doi: 10.1007/s00787-025-02787-6 (PMC12743078; doi:10.1007/s00787-025-02787-6)
Supplement: Supplementary file 2 — Supplementary Material 2 [file 787_2025_2787_MOESM2_ESM.pdf]

Online Resource 2

When stop signal reaction time (SSRT) from the Stop Signal Test (SST) was used as the measure of inhibition, specifically behavioral inhibition, the regressions produced results similar to those obtained with K-KEFS - CW3. SSRT reflects the time required to inhibit a response after a stop signal and was calculated by subtracting the average stop-signal delay from the mean go reaction time, based on the last half of trials. For sample one, the model was significant, $F(5,66)=5.02$, $p<.001$, adjusted $R^2=0.22$. See table S2 (Sample 1) for individual predictors. For sample two, the model was significant, $F(5,79) = 2.77$, $p = .023$, adjusted $R^2 = 0.09$. See table S2 (Sample 2) for individual predictors.

Supplementary Table S2 *Regression Table for Sample 1 & 2 with Social Problems as the Dependent Variable, including SSRT as an Inbibitory Control Measure*

Sample 1					
Variable	Raw B	SE	Std B	***t***	*p*
RTV	2.35	0.98	17.92	2.41	**.019**
RT	(-1.1)	1.02	(-8.36)	(-1.07)	.288
SSRT	0.57	0.84	1.38	0.68	.497
WM (LNS)	(-0.85)	0.27	(-5.47)	(-3.2)	**.002**
Age	1.67	0.76	12.85	2.2	**.031**
Sample 2					
Variable	Raw B	SE	Std B	***t***	*p*
RTV	3.41	1.22	0.32	2.80	**.006**
RT	0.09	1.22	0.01	0.08	.939
SSRT	-9.81	5.75	-0.18	-1.71	.092
WM (DSB)	-0.33	0.37	-0.10	-0.88	.382
Age	-0.70	0.89	-0.09	-0.78	.438

Note. RTV = Reaction time variability; RT = Reaction time; SSRT=Stop Signal reaction time; WM = Working memory; LNS = Letter Number Sequencing (Sample 1); DSB = Digit Span Backward (Sample 2). Raw B = unstandardized beta; Std B = standardized beta.
